# Rapid antimicrobial susceptibility testing and β-lactam-induced cell morphology changes of Gram-negative biological threat pathogens by optical screening

**DOI:** 10.1186/s12866-018-1347-9

**Published:** 2018-12-18

**Authors:** Heather P. McLaughlin, David Sue

**Affiliations:** Laboratory of Preparedness and Response Branch, Division of Preparedness and Emerging Infections, National Center for Emerging and Zoonotic Infectious Diseases, Centers for Disease Control and Prevention, 1600 Clifton Road NE, MS-H17-5, Atlanta, GA 30333 USA

**Keywords:** Antimicrobial susceptibility testing, Cell morphology, Gram-negative biothreat agents

## Abstract

**Background:**

For *Yersinia pestis*, *Burkholderia pseudomallei*, and *Burkholderia mallei*, conventional broth microdilution (BMD) is considered the gold standard for antimicrobial susceptibility testing (AST) and, depending on the species, requires an incubation period of 16–20 h, or 24–48 h according to the Clinical and Laboratory Standards Institute (CLSI) guidelines. After a diagnosis of plague, melioidosis or glanders during an outbreak or after an exposure event, the timely distribution of appropriate antibiotics for treatment or post-exposure prophylaxis of affected populations could reduce mortality rates.

**Results:**

Herein, we developed and evaluated a rapid, automated susceptibility test for these Gram-negative bacterial pathogens based on time-lapse imaging of cells incubating in BMD microtitre drug panels using an optical screening instrument (oCelloScope). In real-time, the instrument screened each inoculated well containing broth with various concentrations of antibiotics published by CLSI for primary testing: ciprofloxacin (CIP), doxycycline (DOX) and gentamicin (GEN) for *Y. pestis*; imipenem (IPM), ceftazidime (CAZ) and DOX for *B. mallei*; and IPM, DOX, CAZ, amoxicillin-clavulanic acid (AMC) and trimethoprim-sulfamethoxazole (SXT) for *B. pseudomallei*. Based on automated growth kinetic data, the time required to accurately determine susceptibility decreased by ≥70% for *Y. pestis* and ≥ 50% for *B. mallei* and *B. pseudomallei* compared to the times required for conventional BMD testing. Susceptibility to GEN, IPM and DOX could be determined in as early as three to six hours. In the presence of CAZ, susceptibility based on instrument-derived growth values could not be determined for the majority of *B. pseudomallei* and *B. mallei* strains tested. Time-lapse video imaging of these cultures revealed that the formation of filaments in the presence of this cephalosporin at inhibitory concentrations was detected as growth. Other β-lactam-induced cell morphology changes, such as the formation of spheroplasts and rapid cell lysis, were also observed and appear to be strain- and antibiotic concentration-dependent.

**Conclusions:**

A rapid, functional AST was developed and real-time video footage captured β-lactam-induced morphologies of wild-type *B. mallei* and *B. pseudomallei* strains in broth. Optical screening reduced the time to results required for AST of three Gram-negative biothreat pathogens using clinically relevant, first-line antibiotics compared to conventional BMD.

**Electronic supplementary material:**

The online version of this article (10.1186/s12866-018-1347-9) contains supplementary material, which is available to authorized users.

## Background

*Yersinia pestis*, *Burkholderia pseudomallei*, and *Burkholderia mallei* are designated Tier 1 biological select agents by the United States Federal Select Agent Program because their deliberate use could pose a serious risk to public health and safety*.* These pathogens are characterized by low infectious doses, high mortality rates, and the ease of dissemination, production and transmission [1, 2]. Both *Y. pestis* (plague) and *B. mallei* (glanders) were weaponized during the twentieth century [3]. Animal herds, including horses and mules, were allegedly infected with *B. mallei* in a deliberate act against Allied forces during World War I [4]. During World War II, a biological and chemical warfare research and development unit of the Japanese military was suspected of intentionally spreading plague-infected fleas and infecting horses, civilians and prisoners of war with glanders [4, 5]. While *Y. pestis*, *B. pseudomallei,* and *B. mallei* are classified as biothreat agents, these Gram-negative pathogens have been more frequently implicated in naturally occurring outbreaks.

As a result of three major plague pandemics, including the Black Death which claimed over 60% of the European population, this disease holds a significant place in human history and has markedly influenced the development of modern civilization [6]. While pandemics have not occurred in recent times, plague is not an eradicated disease and animal hosts still exist on all continents except Australia. Outbreaks such as the 1994 epidemics in western India which led to the evacuation of over half a million residents, and the recent Madagascar outbreak of pneumonic plague, which can spread from person to person, are reminders that plague is a dangerous infectious disease [7–9]. A small number of newly diagnosed plague cases continues to occur in the western United States, but significantly more cases are reported in parts of Africa and Asia. Bubonic plague is the most common form of the disease and is often transmitted by the bite of a flea that has left its infected host. Bacteria from these cases can spread systemically, resulting in secondary septicemia and/or pneumonia [10]. Pneumonic plague can lead to septicemic plague; both types are nearly always fatal without the administration of appropriate antibiotic therapy and care within 24 h of symptom onset [10, 11]. While *Y. pestis* isolates are not intrinsically drug resistant, Russian scientists reported on quinolone-resistant strains [12] and concerns of engineered multi-drug resistant strains have been raised [13, 14]. Furthermore, despite the rarity of drug resistant isolates, both streptomycin- and multidrug-resistant (MDR) clinical isolates have been reported [15–17].

Unlike plague, melioidosis cases are more endemic to Southeast Asia and Australia. Although glanders cases are rare and extremely sporadic, recent naturally occurring equine cases have been documented in Brazil, Pakistan and India [18–20]. Few cases of glanders and melioidosis are reported in the U.S. and with the wide-spectrum clinical manifestations, timely diagnosis and treatment could be challenging [1, 21]. Cases of melioidosis received increased attention after the Vietnam War. Soldiers and pilots experienced variable incubation periods subsequent to pathogen exposure by aerosolization of contaminated soil and water by helicopters [22, 23].

*B. pseudomallei* has a highly variable and evolving genome and has been shown to possess an impressive, inherent array of resistance mechanisms, thus limiting the available antimicrobial agents for therapy [24–26]. Resistance to clinically significant antibiotics has been reported for ceftazidime (CAZ), amoxicillin-clavulanic acid (AMC), trimethoprim-sulfamethoxazole (SXT) and imipenem (IPM) [24, 27, 28]. Moreover, CAZ-resistant variants were reported in vivo in melioidosis patients undergoing CAZ therapy [29, 30]. Without prompt and appropriate antibiotic treatment, mortality rates associated with melioidosis can exceed 40% [26, 31]. *B. mallei* shares morphological and antigenic characteristics with *B. pseudomallei.* Besides aminoglycoside- and macrolide-susceptibility, the reported resistance profiles of *B. pseudomallei* and *B. mallei* strains are similar [32–34]. The mortality rate for pulmonary glanders without treatment has been reported to be 90–95% and as the majority of human cases occurred before antibiotic treatment was available, most infected people perished [35]. Antibiotic therapy for *B. mallei* infections are similar to *B. pseudomallei* and successful treatment has been demonstrated in the few human glanders cases reported since the 1940s [32, 36, 37].

Broth microdilution (BMD) is considered the gold standard method for antimicrobial susceptibly testing (AST) based on the Clinical and Laboratory Standards Institute (CLSI) guidelines, and requires a 16 to 20 h incubation period for *B. pseudomallei* and *B. mallei*, and 24 to 48 h for *Y. pestis* [38]. Gene detection-based methods for AST can reduce the time to results, but the presence of a resistant determinant or gene mutation does not always correspond to phenotypic resistance. Genomic-based analyses rely on prior knowledge of gene(s) or mutation(s) that are responsible for a resistant phenotype and could overlook undescribed, novel resistance. To ensure appropriate antimicrobial agents are deployed in response to an outbreak or other public health emergency event, functional phenotypic susceptibility testing of biothreat agents is essential. Rapid laboratory methods including flow cytometry, bacteriophage amplification and MALDI-TOF mass spectrometry, and bioluminescent reporter phage have been described [39–41]. While these phenotypic methods are less time-consuming compared to the conventional BMD, they can be cost-prohibitive, labor-intensive, and require specialized reagents and training. Rapid characterization of strain(s) implicated in an exposure or release event can also contribute to the public health response. General morphological changes have been described for certain bacterial species in response to β-lactam antibiotics. Some are dependent on drug concentration, binding site specificities, and the number of penicillin-binding protein targets [42, 43].

Previously we developed a simple, rapid, automated antimicrobial susceptibility test for *B. anthracis* using the optical screening instrument the oCelloScope. Time-lapse video imaging of broth culture growth revealed unique chain morphology differences among *B. anthracis* strains [44]. Expanding on this work, herein we evaluated this optical screening method for *Y. pestis, B. pseudomallei*, and *B. mallei*. Real-time video imaging of bacterial-antibiotic combinations in broth culture has revealed β-lactam-induced cell morphology changes of wild-type *B. pseudomallei* and *B. mallei*.

## Materials and methods

### Bacterial strains

Study strains of *Y. pestis*, *B. pseudomallei and B. mallei* are listed with antimicrobial susceptibility profiles and epidemiological data in Table 1. The non-susceptible (NS), virulence-attenuated *Y. pestis* and *B. pseudomallei* strains are excluded from the HHS and USDA Federal Select Agents and Toxins list and were developed as laboratory control strains with the consent and oversight of the CDC Institutional Biosafety Committee. Bacterial strains were stored as glycerol stocks at − 70 °C and then cultured overnight on BD BBL™ Trypticase Soy Agar II with 5% sheep blood plates (SBA) (Fisher Scientific, PA) at 35 °C in ambient air for testing. For *B. pseudomallei* strains Bp82 and JB039, all growth medium was supplemented with 5 μg/ml adenine [45].

**Table 1:** Bacterial strains used in this study and their antimicrobial susceptibility profiles

						Minimal Inhibitory Concentration				
*B. pseudomallei*	Description	Origin	Country	Year	Ref	AMC	CAZ	IPM	SXT	DOX
Bp82	attenuated	Laboratory	USA	2010	[45]	≤ 4/2 (S)	≤ 4 (S)	≤ 2 (S)	≤ 0.5/9.5 (S)	≤ 1 (S)
JB039	attenuated; derivative of Bp82	Laboratory	USA	2016	[49]	32/16 (R)	> 128 (R)	64 (R)	> 32/608 (R)	32 (R)
ATCC 23343	wild-type	Human	n/a	< 1957	[72]	2/1 (S)	1 (S)	0.25 (S)	0.5/9.5 (S)	0.5 (S)
PHLS 14	wild-type	Monkey	Philippines	1990	[73, 74]	4/2 (S)	2 (S)	0.25 (S)	2/38 (S)	1 (S)
B7210	wild-type	Human	Australia	1970	[74, 75]	4/2 (S)	2 (S)	0.5 (S)	1 (S)	2 (S)
Bp1651	wild-type	Human	USA	n/a	[76]	64/32 (R)	> 128 (R)	32 (R)	> 32/608 (R)	16 (R)
*B. mallei*	Description	Origin	Country	Year	Ref	CAZ	IPM	DOX		
ATCC 23344	wild-type	Human	China	1942	[72, 74]	1 (S)	≤ 1 (S)	≤ 0.25 (S)		
NCTC 10260	wild-type	Human	Turkey	1949	[72, 74]	4 (S)	≤ 1 (S)	≤ 0.25 (S)		
Turkey 5	wild-type	Human	Turkey	n/a	[74, 77]	4 (S)	≤ 1 (S)	≤ 0.25 (S)		
KC 1092	wild-type	Mule	Iran	1952	[53, 74, 78]	4 (S)	≤ 1 (S)	≤ 0.25 (S)		
*Y. pestis*	Description	Origin	Country	Year	Ref	GEN	DOX	CIP		
A1122	attenuated	Laboratory	USA	1943	[79]	0.5 (S)	2 (S)	0.03 (S)		
DSJB001	attenuated; derivative of A1122	Laboratory	USA	2016	[49]	32 (R)	> 32 (R)	4 (NS)		
Antiqua	wild-type; biovar Antiqua	Human	Congo	1965	[80, 81]	0.25 (S)	0.5 (S)	0.03 (S)		
Java 9	wild-type; biovar Orientalis	Rat	Indonesia	1957	[80, 82]	0.5 (S)	1 (S)	0.06 (S)		
Nicholisk 41	wild-type; biovar Medievalis	Unknown	China	1940	[80]	0.06 (S)	0.25 (S)	≤ 0.03 (S)		
Angola	wild-type; pestoides group	Unknown	Angola	< 1984	[80, 83]	0.5 (S)	0.25 (S)	0.015 (S)		

Antimicrobial susceptibility profiles were determined by conventional BMD testing based on CLSI guidelines and minimal inhibitory concentrations (MICs) were recorded in the unit of μg/ml. Susceptible (S), non-susceptible (NS), resistant (R)

### Antimicrobials

Antimicrobial agents selected for this study were: gentamicin (GEN), ciprofloxacin (CIP) and doxycycline (DOX) for *Y. pestis*; imipenem (IPM), DOX and ceftazidime (CAZ) for *B. mallei*; and IPM, CAZ, DOX, amoxicillin-clavulanic acid (AMC) and trimethoprim-sulfamethoxazole (SXT) for *B. pseudomallei*. For conventional broth microdilution (BMD) testing, antimicrobial susceptibility panels were prepared in-house with Cation-Adjusted Mueller Hinton broth (CAMHB) as published by CLSI. *Y. pestis* strains were classified as either susceptible or resistant to GEN and DOX, or non-susceptible to CIP based on CLSI designations and conventional BMD results. *B. pseudomallei* strains were classified as susceptible or resistant to AMC, CAZ, IPM, SXT, and DOX. These classifications can be found in Table 1. For antimicrobial susceptibility testing using the oCelloScope instrument, custom-made Sensititre drug panels (Trek Diagnostics, ThermoFisher Scientific, NY) with wells containing desiccated antibiotics were used. For all antimicrobial agents evaluated during oCelloScope-based antimicrobial testing, the lowest concentration tested corresponds to the CLSI breakpoint for susceptibility, followed by two successive two-fold-increasing concentrations. For *Y. pestis*, the antibiotic concentrations tested were 0.25 μg/ml, 0.5 μg/ml and 1 μg/ml CIP, 4 μg/ml, 8 μg/ml and 16 μg/ml DOX, and 4 μg/ml, 8 μg/ml and 16 μg/ml GEN. For *B. mallei* and *B. pseudomallei*, 4 μg/ml, 8 μg/ml and 16 μg/ml IPM, 8 μg/ml, 16 μg/ml and 32 μg/ml CAZ, and 4 μg/ml, 8 μg/ml and 16 μg/ml DOX were tested. For *B. pseudomallei*, concentrations of 8/4 μg/ml, 16/8 μg/ml and 32/16 μg/ml AMC and 2/38 μg/ml, 4/76 μg/ml and 8/152 μg/ml SXT were also tested.

### Biosafety procedures

Laboratory work with the attenuated, select agent-excluded strains was performed by trained personnel wearing personal protective equipment (PPE) in a BSL-2 laboratory. All experiments with wild-type strains were performed in a Class II Type A2 biological safety cabinet (BSC) located in a BSL-3 laboratory registered with the U.S. Federal Select Agent Program by trained personnel wearing PPE including a power air-purifying respirator (PAPR) and protective laboratory clothing [46].

### Imaging of bacterial growth in broth culture by optical screening

Optical screening z-stack images and videos were captured using the oCelloScope instrument (BioSense Solutions ApS, Farum, Denmark) as previously described by Fredborg et al. [47] and McLaughlin et al. [44].

### Susceptibility testing by conventional BMD

Antimicrobial susceptibility profiles and minimal inhibitory concentrations (MIC) outlined in Table 1 were determined by conventional broth microdilution (BMD) following CLSI guidelines [38]. Testing conditions including inoculum, medium and incubation temperature were performed according to these guidelines.

### Susceptibility testing by optical screening

Each bacterial strain was inoculated into Sensititre drug panels by preparing a cell suspension per manufacturer’s recommendations in Cation-Adjusted Mueller Hinton broth (CAMHB) with N-tris(hydroxymethyl) methyl-2-aminoethanesulfonic acid (TES) (CAMHBT; Remel Inc., Lenexa, KS). First, colonies from an overnight SBA culture plate were used to prepare a cell suspension to a turbidity equal to a 0.5 McFarland density standard for each strain. Cell suspensions were diluted in CAMHBT at 1:200 for *Y. pestis* strains and at 1:50 for *B. pseudomallei* and *B. mallei* strains. Drug panel wells were inoculated and then cell suspensions were transferred to a 96-well flat-bottom plate for incubation at 35 °C, as described previously [44]. For the growth kinetic experiments, plates were sealed with a breathable film cover (Breathe-Easy Sealing Membranes, Sigma Aldrich, St. Louis, MO) and placed in the oCelloScope instrument. Growth values were recorded every 20 min for 12 to 18 h, as indicated. General guidelines for antimicrobial susceptibility testing using the oCelloScope instrument are described by Canali et al. [48].

### Analysis of optical screening instrument data

Growth kinetic experiments and image processing were executed using UniExplorer software v. 5.0.3 as described previously [44]. Briefly, the Segmentation and Extraction Surface Area (SESA) normalized algorithm was used to obtain instrument derived growth values. This algorithm identifies bacteria within a scan area based on contrast against the background, and then calculates total bacterial surface area. Microsoft Excel Professional Plus 2013 was used to compile graphical figures. All experiments were performed twice, and figures with growth kinetic data show the mean of triplicate values ± standard deviation (SD) from one representative experiment. The time required to determine susceptibility per strain was defined by statistical analysis of the growth data. A two-tailed t-test (*n* = 3) was used to calculate the statistical significance between a susceptible strain grown in the presence and absence of each antibiotic over time. The earliest times necessary to determine susceptibility are reported as the mean ± standard deviation from duplicate biological experiments and a confidence level of 95% with a *p*-value less than 0.05. Categorical agreement between the optical screening method and conventional BMD for 60 bacterial-antibiotic combinations was used to describe susceptibility results and interpretative errors. Concordance in AST was described as % agreement and discrepancies were reported as % error. In this study, a strain is susceptible by optical screening AST when divergence is observed between growth values in broth without drug and with a concentration of drug two-fold above the CLSI breakpoint for 18 h (*Y. pestis*) or 12 h (*B. pseudomallei* and *B. mallei*) kinetic experiments; a strain is resistant or non-susceptible by optical screening AST when growth values do not diverge. Drugs at concentrations two-fold above the CLSI breakpoints are 8 μg/ml GEN, 8 μg/ml DOX, and 0.5 μg/ml CIP for *Y. pestis*, 8 μg/ml IPM, 4/76 μg/ml SXT, 16 μg/ml CAZ, 16/8 μg/ml AMC, and 8 μg/ml DOX for *B. pseudomallei*, and 8 μg/ml IPM, 8 μg/ml DOX, and 16 μg/ml CAZ for *B. mallei*. A very major error occurs when a strain is susceptible by the optical screening method, but resistant or non-susceptible by conventional BMD. A major error occurs when a strain is resistant or non-susceptible by the optical screening method, but susceptible by conventional BMD. The Segmentation and Extraction of Average Length (SEAL) algorithm was designed to detect filamentation of rod-shaped bacteria based on segmentation extraction of the average bacterial length. SEAL was used to measure the average cell length (μm) of Bp82 and JB039 in the presence and absence of CAZ.

## Results

### Rapid antimicrobial susceptibility testing of attenuated *Y. pestis* and *B. pseudomallei* strains by optical screening

Prior to testing wild-type isolates, we evaluated the oCelloScope optical screening instrument for rapid antimicrobial susceptibility testing of attenuated *Y. pestis* and *B. pseudomallei* strains. Antimicrobial agents used in this study are those described by CLSI for primary testing. MIC values were assessed for every strain by conventional BMD, and results were interpreted based on CLSI breakpoints (Table 1).

**Fig. 1:**
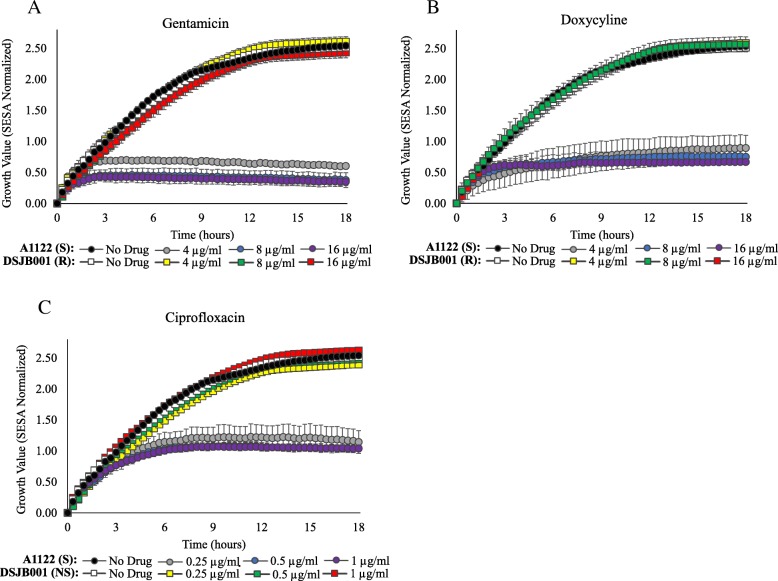
Growth kinetics of attenuated *Y. pestis* strains. A1122 (GEN-S, DOX-S, CIP-S) and DSJB001 (GEN-R, DOX-R, CIP-NS) were evaluated over 18 h at 35 °C in the presence and absence of GEN (**a**), DOX (**b**) and CIP (**c**). Growth was measured by the Segmentation and Extraction of Surface Area (SESA) algorithm. Graphs represent the mean growth value ± standard deviations from three replicate wells

Real-time detection of bacterial growth was measured over 18 h for *Y. pestis* strains in the presence and absence of GEN (Fig. 1a), DOX (Fig. 1b), and CIP (Fig. 1c). In the absence of antibiotics, growth of the susceptible A1122 strain and the resistant/non-susceptible DSJB001 strain in CAMHBT were comparable over time. Growth values recorded in the presence of GEN, DOX, and CIP confirm DSJB001 was not inhibited at the drug concentrations equal to the CLSI breakpoints for susceptibility or at the next two two-fold concentrations above the breakpoints (Fig. 1). These results are consistent with the MIC values of DSJB001, 32 μg/ml GEN, > 32 μg/ml DOX, and 4 μg/ml CIP, in that growth was observed by optical screening in all concentrations of drug tested below the MICs. Growth kinetic experiments also demonstrated the growth inhibition of A1122 in the presence of all drug concentrations tested and growth differences between DSJB001 and A1122 in these conditions can be observed within the first three to six hours. For susceptible *Y. pestis* strains, the time (in hours) required to determine susceptibility was calculated using a two-tailed t-test with a confidence level of 95% (Table 2). These values represent the amount of time in which a statistically significant difference could be measured between the growth of a strain in broth with and without antibiotics. For A1122, susceptibility could be determined the quickest in the presence of GEN after 2.3 ± 0.1, 2.0 ± 0.2, and 1.3 ± 0.7 h for 4, 8, and 16 μg/ml GEN, respectively. For both DOX and CIP, when taking into account SD values, susceptibility of A1122 was resolved in under six hours for all concentrations tested. This is a 75% reduction in time compared to a 24 h conventional BMD test and it is greater than an 87% reduction if the BMD test required 48 h, which is often the case with *Y. pestis*.

**Table 2:**
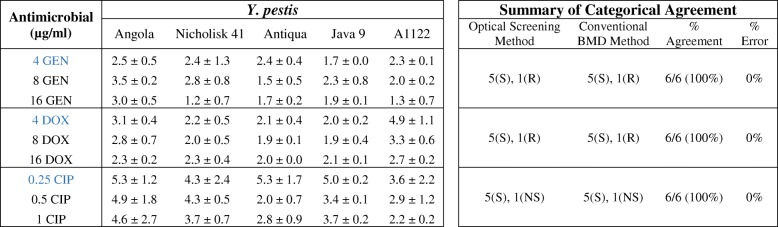
Time (in hours) required to determine susceptibility of susceptible *Y. pestis* strains by optical screening and summary of categorical agreement for AST results by optical screening and conventional BMD for all strains

For susceptible *Y. pestis* strains, the time (in hours) required to determine susceptibility by optical screening is described with a confidence level of 95% (*p*-value ≤0.05). The categorical agreement for susceptibility to GEN, DOX, and CIP for all strains is summarized. Concentrations in blue are equal to the CLSI breakpoint for susceptibility. Strains were grown over an 18 h period and time measurements represent the mean ± standard deviation of duplicate biological experiments (*n* = 3). The number of (S) susceptible, (R) resistant, and (NS) non-susceptible strains, the percentage of categorical agreement and the percentage of error (very major and major) are displayed. DSJB001 was the resistant/non-susceptible strain evaluated

**Fig. 2:**
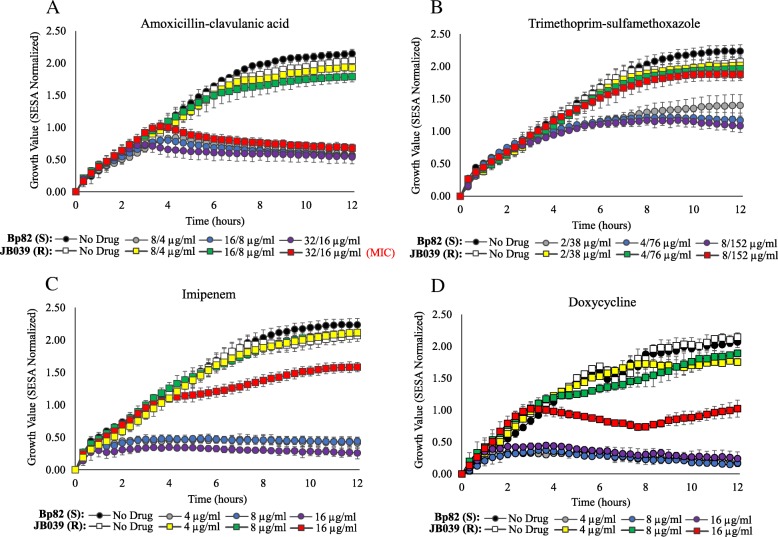
Growth kinetics of attenuated *B. pseudomallei* strains. Susceptible (S) Bp82 and resistant (R) JB039 were evaluated over 12 h at 35 °C in the presence and absence of AMC (**a**), SXT (**b**), IPM (**c**), and DOX (**d**). Growth was measured by the Segmentation and Extraction of Surface Area (SESA) algorithm. Graphs represent the mean growth value ± standard deviations from three replicate wells. The MIC for JB039 in AMC is indicated in red

**Fig. 3:**
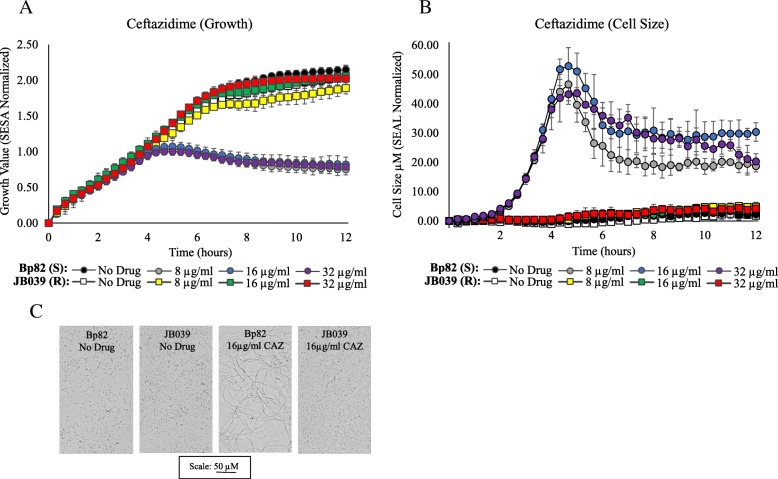
Growth kinetics and cell morphology of attenuated *B. pseudomallei* strains with and without CAZ. Growth (**a**) and cell size (**b**) of the susceptible (S) Bp82 and resistant (R) JB039 strains were evaluated over 12 h at 35 °C. Growth was measured by the Segmentation and Extraction of Surface Area (SESA) algorithm. Cell size was measured by the Segmentation and Extraction of Average Length (SEAL) algorithm. Both graphs represent mean values ± standard deviations from three replicate wells. Optical screen images (**c**) of Bp82 and JB039 were taken of cell suspensions after 6 h in broth containing 16 μg/ml CAZ and in broth alone

For attenuated *B. pseudomallei* strains, growth kinetic experiments were performed over 12 h for the susceptible Bp82 and resistant JB039 strains in the presence and absence of AMC (Fig. 2a), SXT (Fig. 2b), IPM (Fig. 2c), and DOX (Fig. 2d). For JB039, growth response in the presence of each antibiotic at all concentrations tested were consistent with the MIC results obtained for this strain, including inhibition of growth at its MIC of 32/16 μg/ml AMC (Fig. 2a). Partial inhibition of growth was observed for JB039 at the elevated concentrations of 16 μg/ml IPM and 16 μg/ml DOX (Fig. 2c and d). In the presence of drug, differences between growth of JB039 and inhibition of Bp82 could be observed within two to six hours depending on the antibiotic. Differences in growth between these susceptible and resistant *B. pseudomallei* strains was most rapidly detected in the presence of IPM (Fig. 2c) and the time required to determine susceptibly of Bp82 was between 1.6 ± 0.4 and 1.8 ± 0.7 h (Table 3). The longest times required to determine susceptibly of Bp82 were in the presence CAZ which was approximately 6 ± 2 h in the two-fold increasing concentrations from 8 μg/ml (CLSI breakpoint) to 32 μg/ml. Automated growth kinetic experiments showed growth differences between Bp82 and JB039 were observed after approximately 6 h (Fig. 3a). Complementary imaging revealed CAZ-induced filamentation of Bp82 over the first six hours (Fig. 3c) followed by slow cell lysis. The SEAL algorithm can detect filamentation of rod-shaped bacteria and was used to measure the average cell length (μm) of Bp82 and JB039 in the presence and absence of CAZ (Fig. 3b). Over time, cell length remained less than 5 μm for both strains in broth without antibiotic as well as for the resistant JB039 strain in 8 μg/ml, 16 μg/ml, and 32 μg/ml CAZ. However, the average length of Bp82 in the presence of CAZ increased up to 55 μm over the first 6 h during cell filamentation and subsequently decreased as cells began to lyse.

**Table 3:**
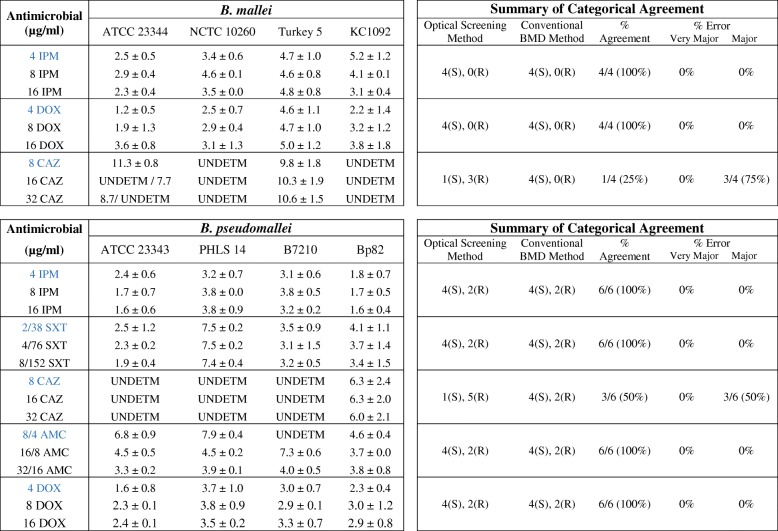
Time (in hours) required to determine susceptibility of susceptible *Burkholderia* strains by optical screening and summary of categorical agreement for AST results by optical screening and conventional BMD results for all strains

For susceptible *Burkholderia* strains, the time (in hours) required to determine susceptibility by optical screening is described with a confidence level of 95% (*p*-value ≤0.05). The categorical agreement for susceptibility to IPM, DOX, CAZ, SXT, and AMC is summarized. Concentrations in blue are equal to the CLSI breakpoint for susceptibility. Strains were grown over a 12 h period and time measurements represent the mean ± standard deviation of duplicate biological experiments (*n* = 3). The time required to predict susceptibility to CAZ and AMC was indicated as undetermined (UNDETM) due to antimicrobial-induced filamentation. The number of (S) susceptible and (R) resistant strains, the percentage of categorical agreement and the percentage of error are displayed. Bp1651 and JB039 were the resistant strains evaluated

### Rapid antimicrobial susceptibility testing of wild-type *Y. pestis*, *B. pseudomallei* and *B. mallei* strains by optical screening

Automated growth kinetic experiments were performed for wild-type *Y. pestis*, *B. pseudomallei* and *B. mallei* strains and times required to determine susceptibility represent the points at which a statistically significant difference was recorded between growth in the presence and absence of clinically relevant antibiotics. Four wild-type *Y. pestis* strains, representing the three biovars (Antiqua, Orientalis and Medievalis) as well as a member of the pestoides group, were tested. Depending on the antibiotic and concentration, testing by optical screening reduced the time required to determine susceptibility by 70 to 93% for all strains when compared to a 24-h conventional BMD test. The most rapid results were obtained for GEN and DOX. The mean time requirements were comparable between the four strains ranging from 1.7 ± 0.0 h for Java 9 in 4 μg/ml GEN to 3.5 ± 0.2 h for Angola in 8 μg/ml GEN (Table 2). Determination of CIP susceptibility was slightly less rapid for all wild-type *Y. pestis* strains with the longest mean time of 5.3 ± 1.7 h recorded for Antiqua. The categorical agreement observed between the optical screening method and conventional BMD was 100% among the four wild-type strains, as well as the two attenuated control strains, in CIP, DOX, and GEN (Table 2). A representative real-time video of *Y. pestis* Angola in broth culture without the presence of antibiotics shows this coccobacilli growing in short chains over time (Additional file 1: Video 1). Similar growth patterns and cell morphologies were recorded for all *Y. pestis* strains grown in broth and no apparent antibiotic-induced cell morphology changes were observed in the presence of CIP, GEN, or DOX (data not shown).


Additional file 1: **Video 1.** Video imaging of *Y. pestis* Angola in broth without drug. (MP4 3041 kb)


For three wild-type *B. pseudomallei* strains, ATCC 23343, PHLS 14 and B7210, susceptibility could be resolved most rapidly in the presence of IPM and DOX (in under five hours for all concentrations tested) which resulted in up to a 75% reduction of time compared to a conventional 16–20 h BMD. Susceptibility to IPM and DOX could be determined in the shortest amount of time for *B. pseudomallei* ATCC 23343 with mean values between 1.6 ± 0.6 and 2.4 ± 0.6 h (Table 3). For SXT testing, the time to determine susceptibility for ATCC 23343 and B7210 was rapid and comparable to those times required for IPM and DOX; however, *B. pseudomallei* PHLS 14 required nearly 8 h in SXT. Complementary video imaging showed slight elongation of PHLS 14 cells in the presence of SXT, which was not observed for ATCC 23343 or B7210 (data not shown). Susceptibility testing of four wild-type *B. mallei* strains in IPM and DOX yielded similar results to those obtained for *B. pseudomallei* strains (Table 3). At concentrations above the breakpoints of 4 μg/ml IPM and DOX, *B. mallei* Turkey 5 required the longest determination times of the *Burkholderia* spp. strains tested; between 4.6 ± 0.8 and 5.0 ± 1.2 h for 8 μg/ml IPM and 16 μg/ml DOX, respectively. For IPM, DOX, SXT, and AMC, there was a 100% categorical agreement between the optical screening and conventional BMD methods among all *B. pseudomallei* and *B. mallei* strains tested (Table 3).

### Ceftazidime susceptibility testing and β-lactam-induced cell morphology changes in *Burkholderia pseudomallei* and *mallei*

**Fig. 4:**
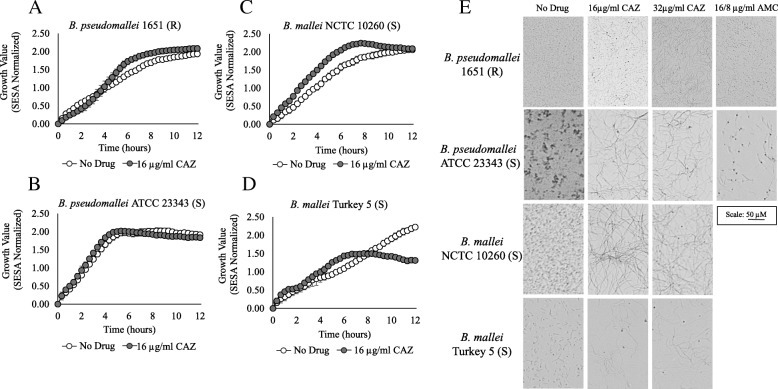
Growth kinetics of wild-type *B. pseudomallei* and *B. mallei* strains. Resistant (R) *B. pseudomallei* 1651 (**a**) and susceptible (S) strains *B. pseudomallei* ATCC 23343 (**b**), *B. mallei* NCTC 10260 (**c**) and *B. mallei* Turkey 5 (**d**) were evaluated over 12 h at 35 °C in broth containing 16 μg/ml CAZ and in broth alone. Optical screen images (**e**) were taken after 8 h in broth alone and in broth containing 16 μg/ml CAZ, 32 μg/ml CAZ and 16/8 μg/ml AMC for *B. pseudomallei* strains and in 16 μg/ml CAZ and 32 μg/ml CAZ for *B. mallei* strains

Instrument-derived growth values could not be used to determine susceptibility to CAZ for the *Burkholderia* strains tested, except *B. mallei* Turkey 5 which required over 10 h (Table 3). Growth kinetic graphs and corresponding images of these susceptible cultures at 8 h revealed the formation of filaments in the presence of CAZ was detected as growth (Fig. 4). Therefore, a statistically significant difference between growth in the presence and absence of this antibiotic could not be established. For instance, growth values obtained for the CAZ-susceptible strains *B. pseudomallei* ATCC 23343 and *B. mallei* NCTC 10260 in the presence of 16 μg/ml CAZ were comparable to those obtained for growth in no drug medium (Fig. 4b and c). These findings are similar to the results from the resistant *B. pseudomallei* 1651 strain in Fig. 4a. As a result, the categorical agreement between optical screening and BMD for CAZ was 25% (75% major error) and 50% (50% major error) for *B. mallei* and *B. pseudomallei*, respectively (Table 3). In contrast, statistically significant differences in later growth values were observed for *B. mallei* Turkey 5 in the presence and absence of CAZ, and for some replicates of ATCC 23344. Imaging showed an initial formation of filaments followed by slow cell lysis after prolonged drug exposure (Fig. 4d).

Based on real-time video imaging of broth cultures, Additional file 2: Table S1 summarizes β-lactam-induced cell morphology changes observed for the drug-susceptible *Burkholderia* strains. Real-time imaging acquired for *B. pseudomallei* strains ATCC 23343, PHLS 14, and B7210, and *B. mallei* strains NCTC 10260 and KC1092 in broth containing CAZ all depict the formation of cell filaments over a 12 h period (Additional file 2: Table S1). Representative videos of *Burkholderia* strains grown in 16 μg/ml CAZ capture this cephalosporin-induced response for *B. pseudomallei* ATCC 23343 (Additional file 3: Video 2) and *B. mallei* NCTC 10260 (Additional file 4: Video 3). For comparative purposes, growth of these two type strains and the resistant *B. pseudomallei* 1651 strain in the absence of antibiotics can be seen in Additional file 5: Video 4, Additional file 6: Video 5 and Additional file 7: Video 6. While strains appeared to aggregate differently during growth in broth without antibiotics, there was no evidence of cell elongation for *Burkholderia* strains tested in drug-free conditions. However, formation of short filaments were observed for the resistant strain *B. pseudomallei* 1651 in 16 and 32 μg/ml CAZ over time and in 16/8 μg/ml AMC during initial logarithmic phase. (Additional file 8: Video 7 and Additional file 9: Video 8 and Fig. 4e**)**.


Additional file 3: **Video 2.** Video imaging of *B. pseudomallei* ATCC 23343 in broth containing 16 μg/ml CAZ. (MP4 1187 kb)



Additional file 4: **Video 3.** Video imaging of *B. mallei* NCTC 10260 in broth containing 16 μg/ml CAZ. (MP4 3072 kb)



Additional file 5: **Video 4.** Video imaging of *B. pseudomallei* ATCC 23343 in broth without drug. (MP4 1822 kb)



Additional file 6: **Video 5.** Video imaging of *B. mallei* NCTC 10260 in broth without drug. (MP4 3031 kb)



Additional file 7: **Video 6.** Video imaging of *B. pseudomallei* 1651 in broth without drug. (MP4 1761 kb)



Additional file 8: **Video 7.** Video imaging of *B. pseudomallei* 1651 in broth containing 16 μg/ml CAZ. (MP4 1986 kb)



Additional file 9: **Video 8.** Video imaging of *B. pseudomallei* 1651 in broth containing 16/8 μg/ml AMC. (MP4 2375 kb)


Observations of *B. pseudomallei* strains in the presence of AMC highlight both concentration- and strain-specific cell morphology changes. For example, the dominant cell morphology of *B. pseudomallei* ATCC 23343 after incubation in 8/4 μg/ml AMC, equal to the CLSI breakpoint for susceptibility, consisted of filaments. Exposure to the two-fold higher concentration of 16/8 μg/ml AMC resulted in the formation of more spheroplasts, and only spheroplasts could be visualized for this strain grown in the next highest concentration of 32/16 μg/ml. These concentration-dependent AMC-induced morphologies of ATCC 23343 captured at 8/4, 16/8, and 32/16 μg/ml can be observed in Additional file 10: Video 9, Additional file 11: Video 10, and Additional file 12: Video 11, respectively. Due to the formation of filaments, AMC susceptibility was undetermined for one strain, *B. pseudomallei* B7210, in one concentration (8/4 μg/ml) (Table 3 and Additional file 2: Table S1). As growth in the presence of IPM resulted in rapid cell lysis for all *B. pseudomallei* and *B. mallei* strains tested, the time required to determine susceptibility in this drug is short. For example, video imaging of *B. mallei* KC1092 in 8 μg/ml IPM captured rapid cell lysis beginning approximately 2 h after drug exposure (Additional file 13: Video 12).


Additional file 10: **Video 9.** Video imaging of *B. pseudomallei* ATCC 23343 in broth containing 8/4 μg/ml AMC. (MP4 1024 kb)



Additional file 11: **Video 10.** Video imaging of *B. pseudomallei* ATCC 23343 in broth containing 16/8 μg/ml AMC. (MP4 662 kb)



Additional file 12: **Video 11.** Video imaging of *B. pseudomallei* ATCC 23343 in broth containing 32/16 μg/ml AMC. (MP4 495 kb)



Additional file 13: **Video 12.** Video imaging of *B. mallei* KC1092 in broth containing 8 μg/ml IPM. (MP4 279 kb)


## Discussion

Optical screening by the oCelloScope instrument reduced the time needed to accurately assess antimicrobial susceptibility of Gram-negative bacterial pathogens *Y. pestis*, *B. pseudomallei* and *B. mallei*. Results are measured in real-time and are available within a few hours, rather than overnight or days. Growth kinetic graphs are plotted automatically by the instrument software in real-time and the data is immediately observable by a laboratorian. Optical screening can detect biological differences in growth rate or cell morphology by comparing the wells of broth culture with and without drug for any strain tested. The predictions of susceptibility by optical screening are based on the growth observations of the same strain in these wells, and do not rely on growth of a control strain. In the presence of antibiotics, the observed growth responses for resistant and susceptible attenuated study strains were consistent with MIC values determined by conventional testing. The laboratory reagents needed for this rapid test and for a conventional BMD are the same, and all drug panels must be inoculated with a cell culture suspension. By contrast, the optical screening method replaces the subjective, visual interpretation of growth/no growth with automated instrument measurements. This method addresses the challenges associated with visually observing BMD plates for bacterial biothreat agents by a laboratory scientist, who must discern growth in small volumes of broth through a BSC wearing the PPE that is necessary for BSL-3 work. Rapid AST for *Y. pestis* by laser light scattering was described previously and requires < 6 h, but the instrument is limited to 16 cuvettes per experiment [49]. Evaluating strain/antibiotic combinations by optical screening in a 96-well plate allows for higher throughput testing and real-time video imaging. Other rapid phenotypic AST methods for *Y. pestis*, including bioluminescent reporter phage-based or flow cytometry methods are more labor-intensive and require subsequent laboratory handling or manipulation of the drug panel post inoculation [50, 51]. After a plague infection is confirmed and the strain has been isolated, rapid and simple susceptibility profiles could be completed in a few hours of testing by optical screening. This is significant as the mortality rate associated with primary pneumonic plague is high when the appropriate antibiotics are not administered within 18–24 h post-symptom onset.

Treatment of melioidosis is divided into two phases that combine the urgency of treating septicemic patients and preventing death (the acute phase) with the need for eliminating persistent disease and preventing relapse (the eradication phase). Several studies reported similarities between the antimicrobial susceptibility profiles of *B. mallei* and *B. pseudomallei* and clinically relevant antibiotics for the treatment of melioidosis, such as CAZ, DOX, and IPM, were effective for the treatment of glanders [32, 33, 52, 53]. Consistent with previous reports, MICs reported here indicate similar antimicrobial activity for both species. Two rapid phenotypic antimicrobial susceptibility tests were previously described for *B. pseudomallei* using laser light scattering technology and bacteriophage amplification/MALDI-TOF MS [39, 49]. Both methods significantly reduced the time required for susceptibility testing compared to conventional methods, but these studies only evaluated the attenuated Bp82 strain and its resistant derivatives. As a rapid AST method for *B. mallei* has not yet been described in the literature, this work represents the first rapid method for wild-type strains of *B. pseudomallei* and *B. mallei*. With the exception of CAZ, the time required to accurately determine susceptibly decreased by at least half for *B. mallei* and *B. pseudomallei* compared to the times required for conventional BMD testing. The slowest susceptibility result based on the optical screening data obtained for *Burkholderia* strains tested in both IPM and DOX was acquired 9+ hours faster than the earliest BMD result would be available (16 h). The rapid results could considerably expedite administration of proper antibiotic therapy during an outbreak or after an exposure event.

Real-time video imaging captured by the oCelloScope allows visualization of bacterial growth simultaneously while performing AST. In a previous study using this optical screening technology, susceptibility of *B. anthracis* could be rapidly determined in ≤4 h and unexpected differences in growth characteristics were observed among strains in the presence and absence of penicillin [44]. Three distinct morphotypes of *B. anthracis* were recorded for replicating cells in broth culture in the absence of antibiotics and some strains took on a crinkled type of morphology in the presence of penicillin. Here, strains of *B. pseudomallei* also appeared to aggregate differently during growth in broth without antibiotics. This highlights the importance of evaluating an assay with a diverse set of isolates. While the *B. mallei* and *Y. pestis* strains selected for this study represent diverse origins and biovars, no differences in cell morphologies were observed among strains during growth in the absence of antibiotics.

Certain Gram-negative bacteria exhibit well-defined morphological changes in response to β-lactam antibiotics including rapid cell lysis, formation of cell wall-deficient spherical cells or filaments [42, 43]. β-lactams covalently bind to penicillin-binding proteins (PBPs) inhibiting the final stage of peptidoglycan (PG) synthesis. While several PBPs are required for PG formation, cell elongation and maintenance of the characteristic Gram-negative rod shape, inactivation of specific PBPs targeted by β-lactam drugs results in well described morphological changes [54–56]. Previous studies report that inactivation of PBP 1A and 1B leads to rapid cell lysis, inhibition of PBP 2 results in the formation of spheroplasts and disruption of PBP 3 causes the formation of long cell filaments [56–58]. In *Y. pestis*, filament formation resulted from furazlocillin binding to PBP 3, and amdinocillin targeting PBP 2 caused the formation of round cells [59]. No cell morphology changes were observed in our study for *Y. pestis* in the presence of CIP, DOX or GEN, but these clinically relevant antibiotics for treatment of plague are not members of the β-lactam group.

For both *B. pseudomallei* and *B. mallei*, growth differences of susceptible strains in the presence and absence of CAZ could not be determined rapidly by optical screening. In *E. coli* and *P. aeruginosa*, the primary activity of CAZ is against PBP 3, and antimicrobial resistance in *B. pseudomallei* involves the loss of BPSS1219, the gene encoding a PBP 3 [29, 60]. Here, we observed CAZ-induced filamentation for all *Burkholderia* strains tested at the drug concentrations 8, 16, and 32 μg/ml. These concentrations are two to 32 times higher than the MIC values for these study strains. As the majority of strains remained filamentous over the entire 12 h of observation, determination of susceptibility was complicated by elongation of cells being interpreted as growth. Fredborg et al. [61] demonstrated early detection of β-lactam-mediated filamentation in susceptible *E. coli* strains followed by cell lysis in the latter hours using the oCelloScope; a similar phenomenon we observed for our *B. pseudomallei* Bp82 and *B. mallei* Turkey 5 study strains. They also show both antibiotic- and concentration-dependent variations in filamentaion. While antibiotic susceptibility is not directly correlated with filamentation, investigations using additional image analysis algorithms could discern differences in early β-lactam-induced morphology changes and could inform timely administration of appropriate antibiotics.

While there have been no other published accounts of β-lactam-induced cell morphology changes in *B. mallei*, filamentation observed for *B. pseudomallei* strains is consistent with research by Chen et al. [62]. In that study, cell elongation was evident for the susceptible *B. pseudomallei* strain KHW in broth containing CAZ at one quarter the MIC and at up to 16 times the MIC. Chen et al. also recommended exercising caution when reducing or discontinuing dosage upon observations of improvement by a melioidosis patient as filamentation induced by sublethal concentrations of CAZ could be reversed when antibiotics were removed, and reverting bacteria were shown to be resistant [62]. While little is known about the morphology of resistant *B. pseudomallei* and *B. mallei* strains in the presence of antibiotics, we observed slight filamentation of the multidrug resistant Bp1651 strain in the presence of CAZ over time and during the initial logarithmic growth phase in AMC. Similarly, resistant *E. coli* has also been shown to form filaments in lag and initial logarithmic phases in the presence of the β-lactam drug cefotaxime [63]. β-lactam-induced filamentation may be an intrinsic *B. pseudomallei* response regardless of whether the strain is drug resistant or susceptible, although further study is needed.

Amoxicillin selectively binds to PBP 4 in *E.coli*, while clavulanate preferentially binds to PBP 2 in *E. coli* and to PBP 3 in *Streptococcus pneumoniae* [64–66]. Observations of various bacterial cell morphologies have been described in the presence of combination drugs and could be indicative of a synergistic effect of complementary binding to PBPs [67, 68]. *E. coli* cells exposed to a combination of clavulanate and cephalexin exhibited reduced filamentation, emergence of spheroplasts and empty sacculi of lysed bacteria [64]. Here, we demonstrate both concentration- and strain-dependent cell morphologies for *B. pseudomallei* strains in the presence of the combination drug AMC. For all wild-type strains evaluated, filaments were the dominant cell morphology type at the breakpoint for susceptibility, but an increase in spheroplast formation was observed with increasing AMC concentrations. In Gram-negative bacteria, the primary target of IPM is PBP 2, and in the *E. coli* strain MC4100, it was reported that IPM and ceftriaxone also had a greater affinity for PBP 1b compared to other drugs [69, 70]. Inhibition of PBP 2 and PBP 1b by drugs such as IPM results in the formation of spheroplasts and rapid cell lysis which is consistent with time-kill studies for *B. pseudomallei* that demonstrated IPM kills more rapidly compared to other drugs [71]. Likewise, we observed both rapid cell lysis via real-time video imaging and rapid determination of antimicrobial susceptibly for all *Burkholderia* strains evaluated.

Evaluation of these biothreat pathogens by optical screening shortened the time needed to determine antimicrobial susceptibility and revealed distinct growth characteristics in broth culture. Using this method, a limited range of relevant drug concentrations was sufficient to determine susceptibility for the study strains of *Y. pestis*, *B. pseudomallei* and *B. mallei* tested. Here, bacterial-antibiotic combinations were analyzed for categorical agreement between AST results obtained by optical screening and conventional BMD. With the exception of CAZ, the categorical agreement was 100% for all drugs tested among the study stains. The rapid AST method described here also reduces the number of steps where pathogenic cultures are handled by a laboratorian which can improve biosafety. In contrast to AST methods that rely on endpoint growth assessments, optical screening measurements are collected in real-time and can be retrieved indefinitely as primary data files. Biosecurity and containment constraints as well as the limited number of resistant strains available make it more challenging to assess rapid ASTs for bacterial biothreat agents. Future studies to evaluate additional strains would aid in assessing the performance characteristics of this method. Real-time video imaging of bacterial-antibiotic combinations has captured β-lactam-induced cell morphology changes in wild-type *B. pseudomallei* and *B. mallei* strains. These cell changes are consistent with the well-defined morphological observations for other Gram-negative pathogens. A better understanding of these responsive morphologies and of β-lactam PBP targets in *B. pseudomallei* and *B. mallei* could contribute to development of improved phenotypic and genotypic susceptibility testing. Observation of a well-characterized cell morphology in combination with a rapid genetic test that can confirm the absence of a specific PBP targeted by a clinically relevant antibiotic may better inform the selection of therapeutics. An understanding of cell morphology changes in *B. pseudomallei* and *B. mallei* strains could reveal characteristics that contribute to more meaningful clinical decisions or by providing critical strain-specific information for the epidemiological investigation.

## Conclusions

For plague, melioidosis, and glanders, the prompt treatment of patients with the appropriate antimicrobial agents could reduce mortality rates. Optical screening can rapidly and accurately assess antimicrobial susceptibility using data collected in real-time for *Y. pestis*, *B. pseudomallei* and *B. mallei*. Here we demonstrate a rapid, functional AST and show real-time time video footage of β-lactam-induced morphologies of wild-type *B. mallei* and *B. pseudomallei* strains in broth. Optical screening reduced the testing time required for AST of three Gram-negative biothreat pathogens using clinically relevant, first-line antibiotics compared to conventional BMD.

## Additional files

Additional file 2: **Table S1.** β-lactam-induced cell morphology changes in susceptible *Burkholderia* strains. (DOCX 22 kb)

## Abbreviations

AMC: Amoxicillin-clavulanic acid
AST: Antimicrobial susceptibility test
BMBL: Biosafety in Microbiological and Biomedical Laboratories
BMD: Broth microdilution
BSC: Biological safety cabinet
CAMHB: Cation-adjusted Mueller Hinton broth
CAZ: Ceftazidime
CIP: Ciprofloxacin
CLSI: Clinical and Laboratory Standards Institute
DOX: Doxycycline
GEN: Gentamicin
IPM: Imipenem
MALDI-TOF MS: Matrix assisted laser desorption ionization-time of flight, mass spectrometry
MDR: Multidrug-resistant
MIC: Minimal inhibitory concentration
NS: Non-susceptible
PAPR: Power air-purifying respirator
PBPs: Penicillin-binding proteins
PPE: Personal protective equipment
SBA: Trypticase Soy Agar II with 5% sheep blood
SD: Standard deviation
SEAL: Segmentation and Extraction of Average Length
SESA: Segmentation and Extraction Surface Area
SXT: Trimethoprim-sulfamethoxazole
TES: N-tris(hydroxymethyl) methyl-2-aminoethanesulfonic acid
