# Supplementary material for: Rapid antimicrobial susceptibility testing and β-lactam-induced cell morphology changes of Gram-negative biological threat pathogens by optical screening
Source: BMC Microbiol. 2018 Dec 18;18:218. doi: 10.1186/s12866-018-1347-9 (PMC6299660; doi:10.1186/s12866-018-1347-9)
Supplement: Supplementary file 13 — Table S1. β-lactam-induced cell morphology changes in susceptible Burkholderia strains. (DOCX 22 kb) [file 12866_2018_1347_MOESM2_ESM.docx]

Supplementary Table 1. β-lactam-induced cell morphology changes in susceptible *Burkholderia* strains.

|  | ***B. pseudomallei*** | | | | ***B. mallei*** | | | |
| --- | --- | --- | --- | --- | --- | --- | --- | --- |
| **Antimicrobial** (µg/ml) | ATCC 23343 | PHLS 14 | B7210 | Bp82 | ATCC 23344 | NCTC 10260 | Turkey 5 | KC1092 |
| 4, 8, 16 IPM | rapid cell lysis | rapid cell lysis | rapid cell lysis | rapid cell lysis | rapid cell lysis | 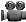rapid cell lysis | rapid cell lysis | rapid cell lysis |
| 8 CAZ | filaments | filaments | filaments | filaments, slow cell lysis | filaments, slow cell lysis | filaments | filaments, slow cell lysis | filaments |
| 16 CAZ | 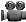filaments | filaments | filaments | filaments, slow cell lysis | filaments,  slow cell lysis | 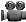filaments | filaments,  slow cell lysis | filaments |
| 32 CAZ | filaments | filaments | filaments | filaments,  slow cell lysis | filaments,  slow cell lysis | filaments | filaments,  slow cell lysis | filaments |
| 8/4 AMC | 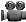filaments (D), spheroplasts slow cell lysis | filaments (D),  spheroplasts  slow cell lysis | filaments | spheroplasts |  |  |  |  |
| 16/8 AMC | 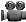spheroplasts (D), filaments slow cell lysis | spheroplasts | filaments (D), spheroplasts slow cell lysis | spheroplasts |  |  |  |  |
| 32/16 AMC | 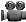spheroplasts | spheroplasts | spheroplasts,  slow cell lysis | spheroplasts |  |  |  |  |


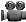
(D) Dominant morphology and ( ) available real-time videos.
